# An immunoassay that distinguishes real neuromyelitis optica signals from a labeling detected in patients receiving natalizumab

**DOI:** 10.1186/1471-2377-14-139

**Published:** 2014-07-01

**Authors:** Ismael Sánchez Gomar, María Díaz Sánchez, Antonio José Uclés Sánchez, José Luis Casado Chocán, Reposo Ramírez-Lorca, Ana Serna, Javier Villadiego, Juan José Toledo-Aral, Miriam Echevarría

**Affiliations:** 1 Instituto de Biomedicina de Sevilla (IBiS), Hospital Universitario Virgen del Rocío/CSIC/Universidad de Sevilla, Av. Manuel Siurot s/n, Seville 41013, Spain; 2 Unidad de Gestión Clínica de Neurociencias, Servicio de Neurología del Hospital Universitario Virgen del Rocío, Seville, Spain; 3 Centro de Investigación Biomédica en Red sobre Enfermedades Neurodegenerativas (CIBERNED), Instituto de Salud Carlos III, Madrid, Spain; 4 Centro de Investigación Biomédica en Red sobre Enfermedades Respiratorias (CIBERES), Instituto de Salud Carlos III, Madrid, Spain

**Keywords:** AQP4-EGFP, NMO-IgG, HEK cells, Natalizumab, Immunohistochemistry

## Abstract

**Background:**

Cell-based assays for neuromyelitis optica (NMO) diagnosis are the most sensitive and specific methods to detect anti-aquaporin 4 (AQP4) antibodies in serum, but some improvements in their quantitative and specificity capacities would be desirable. Thus the aim of the present work was to develop a sensitive quantitative method for detection of anti-AQP4 antibodies that allows clear diagnosis of NMO and distinction of false labeling produced by natalizumab treatment.

**Methods:**

Sera from 167 individuals, patients diagnosed with NMO (16), multiple sclerosis (85), optic neuritis (24), idiopathic myelitis (21), or other neurological disorders (13) and healthy controls (8), were used as the primary antibody in an immunofluorescence assay on HEK cells transfected with the M23 isoform of human AQP4 fused with enhanced green fluorescent protein. Cells used were freshly transfected or stored frozen and then thawed just before adding the serum.

**Results:**

Microscopic observation and fluorescence quantification produced similar results in fresh and frozen samples. Serum samples from patients diagnosed with NMO were 100% positive for anti-AQP4 antibodies, while all the other sera were negative. Using serum from patients treated with natalizumab, a small and unspecific fluorescent signal was produced from all HEK cells, regardless of AQP4 expression.

**Conclusions:**

Our cell-based double-label fluorescence immunoassay protocol significantly increases the signal specificity and reduces false diagnosis of NMO patients, especially in those receiving natalizumab treatment. Frozen pretreated cells allow faster detection of anti-AQP4 antibodies.

## Background

Neuromyelitis optica (NMO) is an inflammatory demyelinating disease of the central nervous system (CNS) that primarily affects the optic nerves and spinal cord [1,2]. Although for long time it was considered a variant of multiple sclerosis (MS), new pathological and serological tests have helped to identify the disorder as a different disease [3]. Lennon and colleagues [4] provided the main evidence for this distinction when they discovered specific immunoglobulins in the serum of NMO patients (NMO-IgG) that were usually absent in classical forms of MS. The antigen recognized for NMO-IgG is aquaporin-4 (AQP4), the most abundantly expressed aquaporin in the CNS [4-8], highly localized in astrocyte membranes facing blood vessel capillaries and in ependymal cells that line the cerebrospinal fluid-filled ventricles and layer of the meninges surrounding the brain and spinal cord [7]. Recent studies have found convincing evidence of a direct involvement of AQP4 autoantibodies in the development of NMO disease [5,9-11]. Magnetic resonance imaging (MRI) in NMO patients indicates that most affected areas coincide with those with higher AQP4 expression [5]. Histopathological lesions observed in the CNS on postmortem show disappearance of AQP4 and deposition of immunoglobulins and products of complement activation in a vasculocentric pattern that coincides with the normal distribution of AQP4 [5,12,13]. Protocols commonly used for NMO diagnosis include MRI studies that are able to identify longitudinally extensive spinal cord lesions extending over three vertebral segments [14,15], with optic nerve involvement and brain lesions in areas of high AQP4 expression [14]. However, the discovery that anti-AQP4 IgG antibodies were present in serum of patients with NMO [4] has revolutionized the diagnosis criteria for this disease and allows more specific treatments that may help reduce the frequency of new relapses.

At least five different methods have been described for detection of anti-AQP4 antibodies in serum of patients [16-25]. Some approaches involve incubation of the serum with mouse brain slices and the signal, well fluorescent or peroxidase, comes from a secondary antibody that recognizes the AQP4 IgG bound to AQP4 [3,4,16,20]. Other assays allow the detection of antibodies by incubation of serum with extracts in which AQP4 is labeled with either radioactive or fluorescent tags prior to the precipitation step [20,21]; and also enzyme-linked immunosorbent assays are being used to detect AQP4 antibodies in patient serum [23,24]. Finally, a cell-based assay initially described as proof of the identification of AQP4 as specific antigen target in NMO positive serum, is nowadays extensively used for routine diagnosis [21,25].

In the present work, we adapted this last method, developing a protocol that combines expression of an AQP4-enhanced green fluorescent protein (EGFP) with the use of a red fluorescent goat anti-human secondary antibody. By this double labeling, we obtained a method with extremely high sensitivity and specificity for identifying NMO positive patients and that additionally enables quantitative comparison of antibody levels in sera samples tested at the same time. Moreover, the high signal specificity of the method we describe here allows false signals to be distinguished from those produced by sera from patients treated with natalizumab.

## Methods

### Subjects and serum recollection

The study population includes 167 subjects (116 female) classified into 6 groups based on their medical diagnosis (Table 1). Group 1 comprised 16 patients with NMO according to the current diagnostic criteria [2]. Group 2 consisted of 85 patients with MS according to 2010-revised McDonald criteria [26]. In relation to the MS course, we further divided this group into 72 patients with relapsing-remitting MS, 10 with primary progressive MS and 3 with a secondary progressive form of the disease. Group 3 was composed of 24 patients with optic neuritis. Twenty-one patients with myelitis formed group 4. Concerning the length of the spinal cord lesion, we distinguished between patients whose lesions extended more than three vertebral segments (longitudinally-extensive myelitis) and those with shorter lesions (9 patients in each group respectively). In addition, we categorized the patients of groups 3 and 4 depending whether they had had an isolated episode or multiple recurrences. Group 5 comprised 13 patients with other neurological disorders (2 patients with myelitis associated with lupus, 1 patient with optic neuritis associated with Sjögren syndrome, 2 patients with multifocal motor neuropathy, 1 patient with chronic inflammatory demyelinating polyneuropathy, 2 patients with spinal infarction, and 5 patients with ischemic optic neuropathy). Finally, group 6 was composed of 8 healthy controls. Besides the MRI scans and the cerebrospinal fluid analysis, peripheral blood exams were conducted for all patients in order to exclude alternative etiologies and confirm their final diagnosis. These exams included blood cell counts, assessment of erythrocyte sedimentation rate, and levels of vitamins, thyroid hormones, long chain fatty acids, angiotensin converting enzyme as well as basic biochemical parameters, and immunological and serological tests. After collection, blood was centrifuged at 2500 rpm (15 min, 4°C) and the serum kept at -80°C until use.

**Table 1:** Demographic and clinical variables of patients

**Diagnosis**	**Number of patients**	**Gender (Female/Male)**	**Mean age at inclusion +/- SD (range)**
**Group 1: NMO**	16	13/3	55.02 +/- 9.80
			(40-63)
**Group 2: MS**	85	60/25	39.43 +/-9.40
			(18-76)
- Relapsing-remitting MS	72		
- Secondary progressive MS	3		
- Primary progressive MS	10		
**Group 3: idiopathic ON**	24	17/7	35.01 +/- 11.39
			(14-62)
-Isolated episode	15		
-Recurrent idiopathic ON	9		
**Group 4: idiopathic myelitis**	21	14/7	46.30 +/- 14.27
			(21-68)
-Isolated episode:	18		
> 3 vertebral segments	9		
≤ 3 vertebral segments	9		
-Recurrent idiopathic myelitis	3		
**Group 5: other neurological disorders**	13	7/6	50.45 +/- 6.11
			(42-59)
-Myelitis associated with lupus	2		
-ON associated with Sjögren syndrome	1		
-Multifocal motor neuropathy	2		
-CIDP	1		
-Spinal infarction	2		
-Ischemic optic neuropathy	5		
**Group 6: healthy controls**	8	5/3	34.45 +/- 8.31
			(27-50)

SD; standard deviation; NMO: neuromyelitis; MS: multiple sclerosis; ON: optic neuritis; CIDP: chronic inflammatory demyelinating polyneuropathy).

The patients (Groups 1-5) and the healthy controls (Group 6) were recruited by the Service of Neurology at the Virgen del Rocío University Hospital and the IBiS, respectively. For all participants, written consent was obtained before their inclusion in the study, and demographic and clinical variables were recorded including gender, age at inclusion in the study, clinical diagnosis and treatments received (Table 1). The study counted with the approval of The Ethics Committee of The University Hospital Virgen del Rocío (HUVR), with the registration number 14/2010.

### Plasmid construction, cell culture and cell transfection

The M23 isoform of human AQP4 was amplified by polymerase chain reaction (PCR) from the commercial vector pDNR-LIB cDNA using specific primers (Table 2) and cloned into pEGFP-N1 (both Takara Bio Europe/Clontech, France), for transfection and expression in the HEK293T cell line. This allowed synthesis of an AQP4-fluorescent fusion protein in which the EGFP was bound to AQP4 by its carboxyl end. HEK cells were cultured in Dulbecco's modified Eagle's medium with 10% fetal bovine serum and 1% penicillin/streptomycin (37°C, 5% CO_2_). Cells (2 × 10^5^) were seeded in 35-mm dishes for transfection with Lipofectamine 2000 (Invitrogen) as previously described [27]. Transfected cells were maintained for 25-30 passages until the fluorescence signal of AQP4 expression decreased to below 90%.

**Table 2:** Primers for PCR amplification of full-length human aquaporin 4 (hAQP4)

**Gene**	**Primer sequences**
**hAQP4-M23**	**Forward :** 5′-ACTC*CTCGAG*GGCGGTGGGGTAAGTGTGGAC -3′
	**Reverse:** 5′-ACTC*CCCGGG*AATGGGTGGAAGGAAATCTGA -3′

Primers were designed using the Primer Express software (Applied-Biosystems). Sequences of restriction enzymes used for cloning purpose are indicated: *CTCGAG:* Xho1 and *CCCGGG:* Xma1.

### Immunofluorescence assay and signal quantification

**Figure 1:**
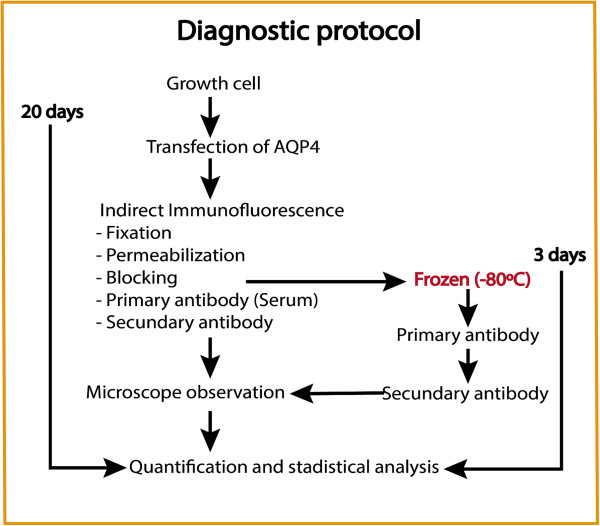
**Optimization of a standard protocol for NMO diagnosis based on indirect immunofluorescence of cells expressing AQP4.** The normal cell-based assay -using AQP4 as the antigen target in NMO-IgG detection- starts with culturing the cells and transfecting them with an AQP4 vector to achieve expression of the protein, before initiating the immunocytochemistry protocol itself. Then, standard procedures of fixation, permeabilization and blocking are performed before incubation with the primary and secondary antibodies, in a protocol that would take approximately 20 days. On the other hand, frozen cells overexpressing AQP4 can be stored, for a considerable time, at -80°C just after the blocking step, and be used for continuation of the immune protocol on receiving a patient’s serum, producing equivalent results in only 3 days.

Based on previous methods [18], we developed a simple quantitative protocol that would allow us to follow changes in anti-AQP4 antibodies in the serum of patients. We started by performing a double blind assay in which serum from 20 patients kindly provided by Dr. A. Saiz [16] were re-tested with our assay confirming the original diagnosis in all cases. Then, immunofluorescence assays were performed on cells plated and transfected 24 h before the basic assay (without freezing) or/and cells identically treated up to the blocking step, but stored at -80°C (frozen) until incubation with the serum (short assay). The steps of the two immunofluorescence assays are shown in Figure 1. Cells were washed with phosphate buffered saline (PBS)-Ca^++^-Mg^++^ and fixed with 3% paraformaldehyde in PBS for 5 min, followed by four washes in PBS. They were then permeabilized with triton 0.1% in PBS (PBTx, 5 min) and blocked using 10% FCS with 1 mg/ml BSA in PBTx (1 h). For the protocol using frozen cells, 10% dimethyl sulfoxide (DMSO) was added to the blocking solution before sealing the plate with parafilm. Afterwards, cells were incubated for 1 h with the patient’s serum (1:50 dilution), followed by another hour of incubation with Alexa Fluor 568 goat anti-human secondary antibody. Nuclei were stained with 4’, 6’-diamidino-2-phenylindole (DAPI, 1:1000). A Leica DM IRBE confocal microscope was used to take five random images (40×) per sample and NIH ImageJ software used for densitometric analysis of the fluorescence.

### RT-qPCR amplification of the integrin α4 chain

RNA was extracted from HEK cells with Trizol (Invitrogen) and reverse transcribed using the SuperScript II Reverse Transcriptase kit (Invitrogen). Quantitative PCR analysis was performed in an ABI Prism 7500 Sequence Detection System using SYBR Green PCR Master mix (both from Applied Biosystems, Warrington, UK) and conditions recommended by the manufacturer. Primer sequences are indicated in Table 3. Cyclophilin and β-actin were used as housekeeping genes to normalize for differences in RNA input amounts, and data was represented with respect to wild-type HEK cells. Melting curve analysis showed a single sharp peak at the expected melting temperature for all samples.

**Table 3:** Primers for qRT-PCR analysis of integrin α4

**Gene**	**Forward primer sequence**	**Reverse primer sequence**
α4 INTEG	5′- GGCAAGGAAGTTCCAGGTTACAT -3′	5′- ATGCTTCCTGTAATCACGTCAGAA -3′
CYCLO	5′- ATGGCAAATGCTGGACCAAA-3′	5′- CATGTCGTCCCAGTTGGTAACA-3′
β-ACTIN	5′- AGGCCAACCGTGAAAAGATG-3′	5′- GCCTGGATGGCTACGTACATG-3′

Primers were designed using the Primer Express software (Applied Biosystems).

### Statistical analysis

Data are presented as mean ± standard error of the mean, and all statistical analyses were conducted using the IBM SPSS Statistics (IBM Corp., Armonk, NY), version 19.0. Data with a non-normal distribution were analyzed using analysis of variance (ANOVA) for nonparametric data, using the Kruskal-Wallis H or Mann-Whitney U tests for two or more than two groups, respectively.

## Results

### Optimization of an immunofluorescence protocol for detection of anti-AQP4 antibodies

**Figure 2:**
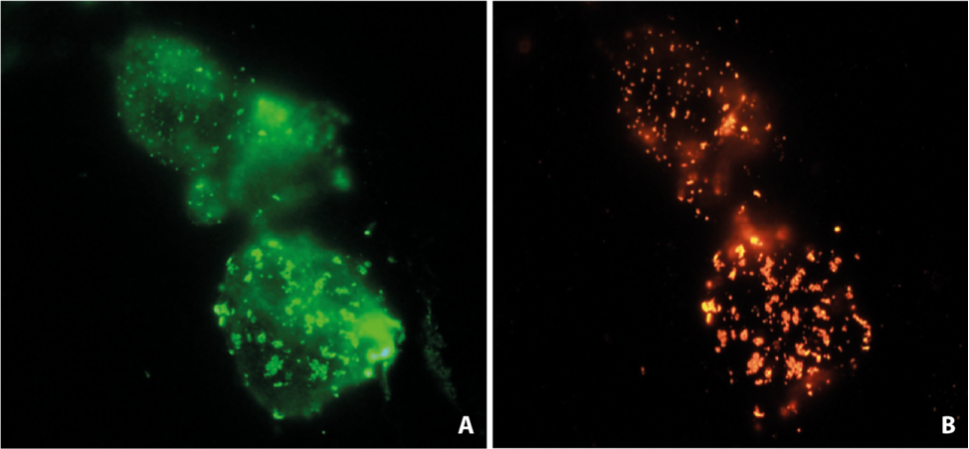
**Immunofluorescence assay in HEK cells.** Co-localization of fluorescent labeling was detected in cells expressing AQP4-EGFP when a direct fluorescence signal was observed **(A)** or when serum from an NMO patient was used as the primary antibody (NMO-IgG) in an immunoassay **(B)**.

The fluorescent protein (human AQP4-EGFP) allowed direct visualization of the AQP4 cellular distribution in HEK-transfected cells. Transfection efficiency was always above 90%. Direct green fluorescence labeling of AQP4-expressing cells showed a spotty distribution (Figure 2A), resembling patches of the orthogonal array pattern characteristic of this protein [28,29]. Indirect immunofluorescence reaction of cells with a positive control serum (C+) showed an almost identical distribution of the red fluorescent labeling produced by the anti-human-IgG secondary antibody (Figure 2B) confirming the presence of anti-AQP4 antibodies in the serum.

**Figure 3:**
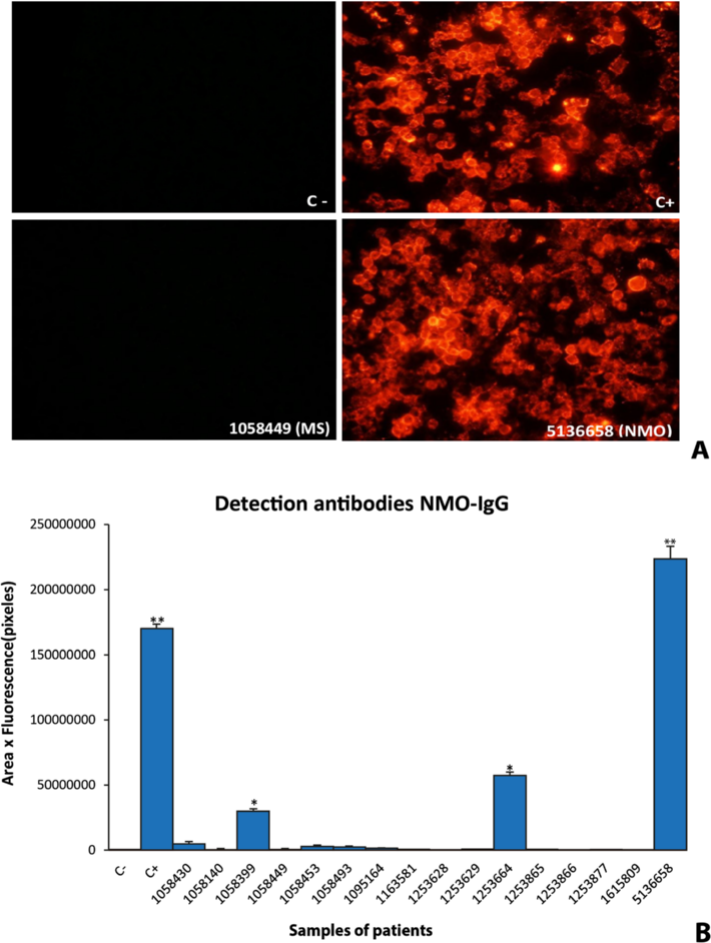
**Development of quantitative analysis from immunofluorescence assay.** Serum from diverse patients were used as primary antibody in an immunofluorescence assay **(A)**. Quantification of fluorescence resulted from the densitometric analysis of fluorescence intensity using the NIH ImageJ software taking in account the fluorescent area **(B)**. C+, corresponds to a positive control serum in which NMO-IgG antibodies had previously been confirmed, and C-, corresponds to a negative control serum in which the antibodies were absent. Sera from different patients appear indicated with an identifying reference number. Significant differences (* p ≤ 0.05 and ** p ≤ 0.01) with respect to C- serum are indicated. Error bars are ± SEM (n = 5).

Using this basic protocol, we then analyzed serum from the 167 individuals included in this study. Representative examples of fluorescence photographs of four different serum samples are shown in Figure 3A and the quantitative analysis of 16 samples is presented in Figure 3B. Fluorescence is not visible in either the negative control (C-) serum or in serum from a MS patient (Figure 3A), consistent with detection of weak fluorescence signals from both types of sera (Figure 3B). By contrast, strong fluorescence signals (p < 0.01) were observed both in the positive control (C+) serum and serum from a newly diagnosed NMO patient (sample # 5136658). A high fluorescence value in these two types of sera again evidenced perfect correspondence between the qualitative microscopic observation and the numerical quantification from the densitometric analysis. These findings confirmed the direct correlation between detection of anti-AQP4 antibodies and a clinical diagnosis of NMO. Moreover, NMO antibodies were not detected in any of the patients diagnosed with pathologies other than NMO or in healthy controls. Quantitative analysis from immunofluorescence assay comparing all groups is shown in Additional file 1: Figure S1.

### Comparative analysis of immunofluorescence assay using fresh and frozen cells

**Figure 4:**
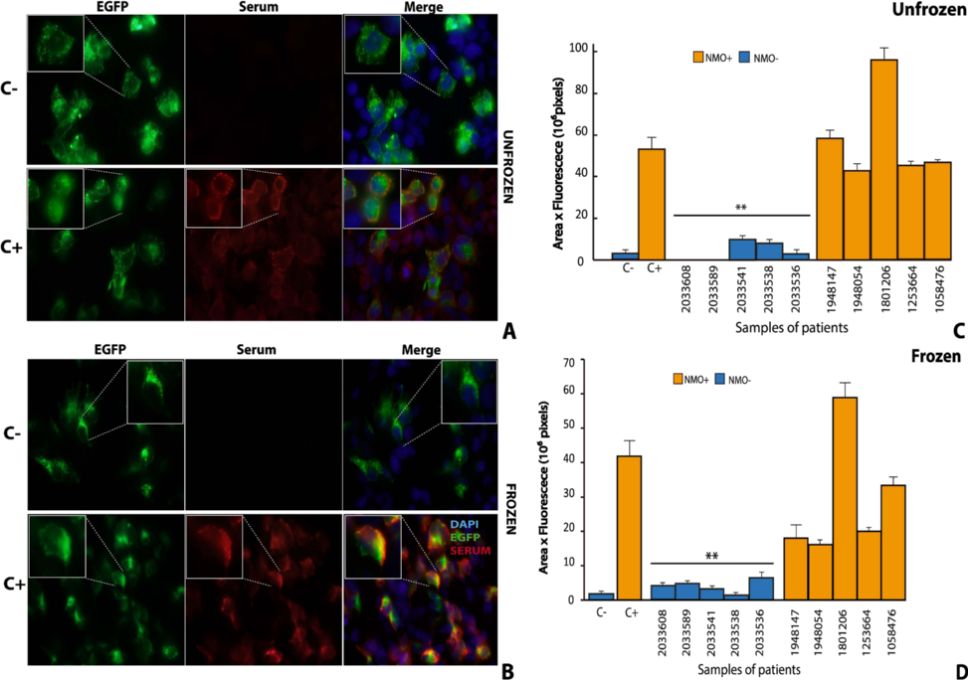
**Comparative analysis of cell immunohistochemistry results obtained using fresh or frozen cells. A** and **C**, correspond to experiments done on fresh cells; and **B** and **D**, on frozen cells. In **A** and **B**, the fluorescence signal from HEK cells expressing AQP4-EGFP is shown in green (left column), and that from the secondary antibody bound to the NMO-IgG in red (middle column), while the merged image of both fluorescent signals is shown in yellow (right column). Large magnification images are boxed. Two patients’ serum were used in the analysis; C+, in which NMO-IgG antibodies had been confirmed previously, and C-, in which the antibodies were absent. **C** and **D** summarize the quantitative analysis. A significant difference (** p ≤ 0.01) with respect to C + serum was obtained as indicated. Error bars are ± SEM (n = 5).

To develop a rapid diagnosis protocol, the basic immunofluorescence assay was performed using in parallel fresh and frozen transfected cells. A representative example of the comparative analysis is shown in Figure 4, where similar results are obtained with the two protocols. The green fluorescent signal detected in either fresh (Figure 4A) or frozen (Figure 4B) cells expressing AQP4-EGFP was similar and so was the fluorescent signal (red) coming from the secondary antibody (anti-IgG) that recognized the anti-AQP4, leading in both cases to similar merged fluorescent images (orange). A summary of the quantitative analysis obtained for 10 different sera, 5 NMO positive and 5 NMO negative, is shown in Figure 4C (fresh) and 4D (frozen). With both protocols, all patients negative for NMO antibodies showed lower levels of fluorescence (** p < 0.01) than those obtained with the C + serum, whereas signals from positive sera were not statistically different to those from C + serum. Overall, less fluorescence was observed when frozen cells were used, but differences between NMO positive and negative samples were qualitatively maintained in both cases.

### Positivity of serum from natalizumab-treated patients

**Figure 5:**
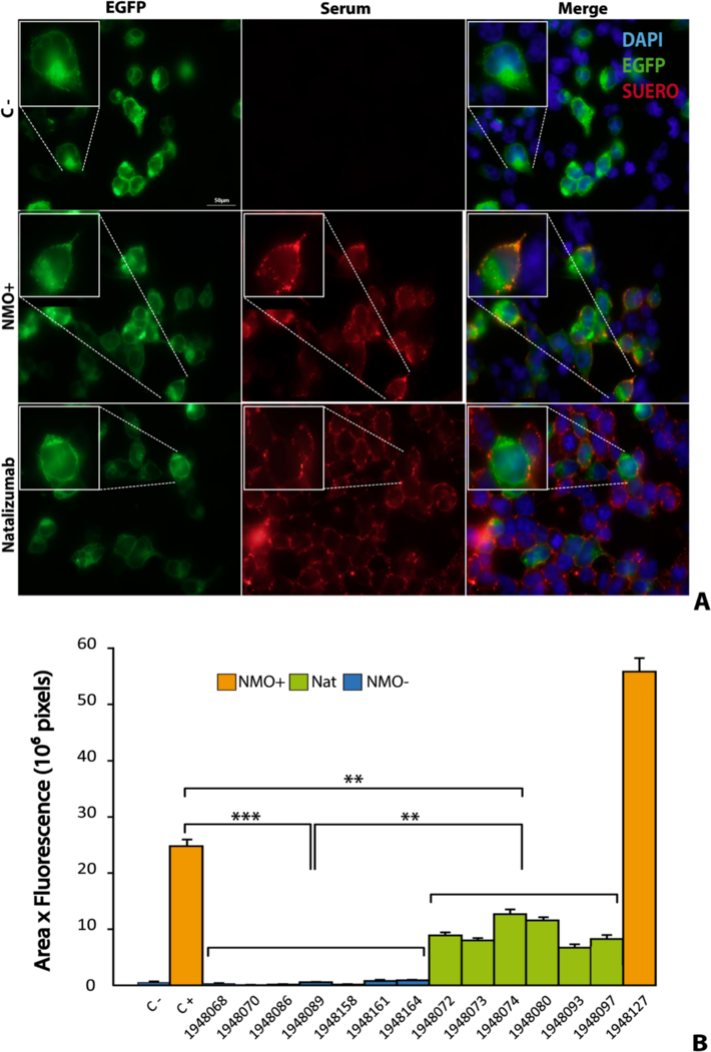
**Positivity of fluorescence labeling with serum from natalizumab-treated patients. A:** Shows fluorescence microphotographs from HEK cells expressing AQP4-EGFP (left column), or the fluorescent signal after the immunoassay (middle column) with patients serum, and the merged image of both fluorescent signals (right column). Serum from three types of patient was used in the analysis: C-, in which NMO-IgG was absent; NMO+, in which NMO-IgG antibodies had been confirmed previously; and “Nat”, in which serum from a patient treated with natalizumab was used. Immunofluorescence signals (red) were observed both in NMO + and Nat, although the merged image confirms exclusive co-localization of red and green labels only in the NMO + case. All HEK cells (DAPI stained) independently of whether they express AQP4 or not show some red fluorescence signal with Nat serum, thereby producing a signal that, though low, was above background (C-) level. **B:** Summarizes the quantitative analysis. Significant statistical differences (** p < 0.01, *** p < 0.001) were observed when signals were compared to C + serum or comparing Nat and NMO- samples (**p < 0.01). Error bars are ± SEM (n = 6-7).

Using the basic immunofluorescence protocol, we discovered a distinctive labeling pattern in serum from a subgroup of patients, who we later confirmed were all on natalizumab or had received this treatment within the 6 months prior to blood sample collection (Table 4). As can be observed in Figure 5A, cells expressing AQP4-EGFP (green, left column) showed a consistently high fluorescent signal, while incubation with the serum of natalizumab-treated patients produced slight fluorescence (red, middle column) detectable across all cells present in the plate, regardless of whether or not they were expressing the AQP4 protein, and NMO + serum exclusively labelled cells with high expression of AQP4 (red, center photograph). Merging fluorescence images (orange, right column) reveals strong overlapping of signals only when NMO + serum was used. By contrast, when natalizumab serum was used fluorescence signals were separated and merged images did not reveal overlapping of the signal over the same cells, evidencing a nonspecific immune signal. In the C- serum, absence of red labeling demonstrated lack of anti-AQP4 IgG. A summary of the quantitative analysis obtained for 14 different sera, 7 NMO negative, 6 with natalizumab labeling and 1 NMO + is represented in Figure 5B. All patients negative for NMO antibodies showed lower levels of fluorescence (*** p < 0.001) than the value for the C + serum. Statistically significant differences were observed between serum from natalizumab-treated patients and C + serum (** p < 0.01) and also between serum from NMO- and natalizumab-treated patients (** p < 0.01).

**Table 4:** Serological data for 167 subjects included in the study

**Natallizumab treatment**	**Number of patients**	**Group**	**False labeling pattern**
**Yes**	20	2	17*
**No**	147	1-6	0

*These patients were on natalizumab at the time blood samples were taken or stopped receiving this drug less than 6 months prior to collection of the blood sample.

## Discussion

Nowadays, the detection of AQP4 autoantibodies (NMO-IgG), as a serum biomarker for NMO, is an essential test for a final diagnosis of this disease. Our initial goal was to develop a simple detection method for use in routine practice and we began by assessing results with immunoassay procedures such as Western blots and direct detection of AQP4 by immunohistochemical analysis. After many trials, only nonspecific results were obtained with both of these approaches, forcing us to explore alternatives and we started to work on a cell-based immunofluorescence assay using transfected cells with high expression of the human AQP4 M23 isoform.

The protocol we have developed and described herein consists of a cell-based double-label fluorescence immunoassay, using two fluorophores with different emission wavelengths: green, to directly visualize the expression of human AQP4 in HEK cells; and red to visualize the antihuman-IgG that binds to AQP4 antibodies in serum. The co-localization of the two fluorescence signals evidences the presence of NMO-IgG in serum and, therefore, represents a specific reaction that confirms the NMO positive diagnosis. Only 16 of all the individuals analyzed had autoantibodies for AQP4 in their serum and therefore had an NMO positive diagnosis. Notably, quantification of fluorescence signals obtained with sera from these 16 patients also revealed high levels of NMO-IgG, markedly higher than levels detected in any other serum sample analyzed. Accordingly, the diagnosis of patients based on serum immune response agreed with the diagnosis reached by neurological examination based on the current diagnostic criteria in 100% of cases [2].

While to our knowledge previously reported assays to detect anti-AQP4 antibodies in NMO rely either on microscopic observation of specific immune signals (visual fluorescence or colored precipitate), or on quantification of colorimetric, fluorescent or radioactive signals [25], the diagnostic assay we present takes into account both localization and magnitude of the fluorescent signal. Combining these features, we have obtained a diagnostic assay with high sensitivity and specificity that would likely reduce false positive results and would improve detection of NMO-IgG when present at low levels. Quantification of the fluorescence intensity indicates NMO-IgG positivity with fluorescence signals that were clearly stronger than those obtained with controls with this assay and the consistency in repeated measurements suggests that it is a reliable and reproducible method. So far, we have not investigated variations in antibodies levels over time, but this is a future application of our method and it is plausible to suppose that the results will have predictive value for disease progression and treatment response.

Results presented here also demonstrate that freezing HEK cells expressing AQP4-EGFP after permeabilization, ready to hybridize with serum as soon as sample arrive at the laboratory, can substantially shorten the standard cell-based fluorescent immunoassay protocol, to about 3 days, without losing sensitivity or specificity. This would accelerate diagnosis from serum samples and is an approach that could potentially be used to develop rapid kits for NMO diagnosis in hospital services where cell culture and cell biology facilities are not easily available.

In a clinical context, another important finding in the present study comes from experiments in which detailed microscopic observation revealed a subgroup of sera in which, after hybridization with AQP4 HEK-transfected cells, a small fluorescent signal was observed from every single cell regardless of whether there was cellular expression of the fluorescent AQP4-EGFP protein. Surprisingly, the sera producing this distinctive and confusing staining pattern, that could lead to misdiagnosis of patients as NMO positive, all came from patients that received natalizumab treatment within the 6 months prior to blood sample collection. Quantification of the fluorescence signals corroborated a positive immune reaction, but although higher than those detected with C- (NMO-IgG (-)), signals were clearly weaker than those obtained with positive control sera (NMO-IgG (+)).

**Figure 6:**
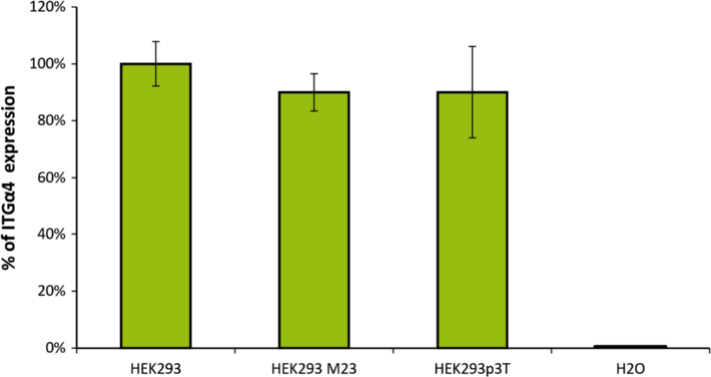
**Detection of α4 chain integrin in HEK cells by RT-qPCR.** Percentage of expression of integrin α4 (ITGα4) was similar in control non-transfected HEK cells (HEK293) or in HEK cells transfected with AQP4 (HEK293M23) or with the empty vector (HEK293p3T). No amplification was observed in the water control sample.

Natalizumab is a recombinant humanized monoclonal antibody that binds to the α4 chain of α4β1 and α4β7 integrins of the very late antigen (VLA)-4 in leucocytes. It blocks leucocyte migration across the blood-brain barrier into the CNS and with that reduces the inflammatory reaction in patients with relapsing-remitting MS [30]. To explain the peculiar false labeling pattern observed when serum from natalizumab-treated patients was used, we hypothesized that natalizumab antibodies were binding to the membrane of HEK cells in an unexpected way and, in turn, being recognized by the red fluorescent antihuman-IgG used in the diagnostic assay. Then, using RT-qPCR, we explored the expression of α4 chain integrin in the HEK cells, and confirmed similar expression levels of this protein in HEK cells whether or not they were transfected with AQP4 (Figure 6). We directly applied an aliquot of concentrated natalizumab (TYSABRI, 20 mg/ml), the same formulation as that used for patient treatment, to AQP4 HEK-transfected cells and confirmed binding of this monoclonal antibody on cells that both did and did not express AQP4 (Additional file 2: Figure S2).

To characterize the diagnostic assay, it was of interest to assess the immune fluorescence-staining pattern in sera obtained from patients who had taken natalizumab but stopped the treatment more than 6 months before the blood test. We found that the fluorescence-staining pattern in these samples was indistinguishable from that obtained with a C- serum, indicating that long periods of time (at least 6 months) allow sufficient reduction in circulating levels of natalizumab in serum for the false labeling pattern to disappear.

Hence, while HEK cells offer very convenient features, such as high transfection efficiency and levels of expression of heterologous human AQP4, a drawback of using these cells in NMO diagnostic assays is also evidenced in the present work: specifically, the expression of integrin proteins in their membrane seems to elicit the binding of natalizumab antibodies that, in turn, can be recognized by human anti-IgG antibodies and result in a false signal for NMO diagnosis. Exploring this cross reactivity of natalizumab in different cell lines would be important to further improve the cell-based double-label fluorescent immunoassay procedure presented in this work. Moreover, a similar response to the cross reactivity observed with natalizumab could also be produced by other immunotherapies, potentially leading to false positive diagnoses of NMO. In view of these results, we should also underline the secondary effects some immunotherapies might have by binding to as yet unknown targets in the human body.

## Conclusions

In conclusion, the use of HEK cells expressing AQP4-EGFP that are stored frozen after the blocking step make it possible to obtain a final result within a few days of receiving serum samples, accelerating the diagnosis of NMO. A limitation was found in the use of HEK cells and may be explained by false signals from the binding of natalizumab antibodies to integrins present in HEK cell membrane that, in turn, can be recognized by human anti-IgG antibodies. Nevertheless, the cell-based double-label fluorescent immunoassay protocol we present here significantly increases the signal specificity and reduces the likelihood of false diagnoses of NMO, especially in patients treated with natalizumab.

## Abbreviations

AQP4: Aquaporin-4; NMO: Neuromyelitis optic; MS: Multiple sclerosis; EGFP: Enhanced green fluorescent protein; IgG: Immunoglobulin G; HEK: Human embryonic kidney cells; MRI: Magnetic resonance imaging; CNS: Central nervous system; RT-qPCR: Reverse transcription quantitative polymerase chain reaction.

## Competing interests

The authors declared that they have no competing interests.

## Authors’ contributions

ME conceived and designed the experiments and wrote the manuscript; ISG, AS, RRL and JV performed the molecular biology experiments; ISG carried out the immunoassays; MDS, AJUS, JLCC recruited and diagnosed the patients and collect information from them; ME, ISG, JJTA, JVD and MDS analyzed the data; ME and JJTA contributed reagents/materials/analysis tools; and all authors read and approved the final manuscript.

## Pre-publication history

The pre-publication history for this paper can be accessed here:

http://www.biomedcentral.com/1471-2377/14/139/prepub

## Supplementary Material

Additional file 1: Figure S1 Quantitative analysis from immunofluorescence assay. Serum from patients were used as primary antibody in an immunofluorescence assay. Quantification of fluorescence resulted from the densitometry analysis of fluorescence level using the NIH ImageJ software taking into account the fluorescent area. C+, corresponds to a positive control serum in which NMO-IgG were previously confirmed, and C-, corresponds with a negative control serum in which the Ab was absent. Quantification of fluorescence signal from serums of the six different groups was averaged together. Significant differences (*** p ≤ 0.001) respect to C- serum are indicated. Error bars are ± s.e.m (n=5). NMO: neuromyelitis; MS: multiple sclerosis; ON: optic neuritis; ND: neurological disorders. Click here for file

Additional file 2: Figure S2 Immunofluorescence assay using serum from natalizumab treated patient vs natalizumab reagent. Green fluorescent labeling was only detected in cells expressing EGFP-AQP4. However similar red fluorescence labeling was observed over all cells (expressing or not AQP4) when serum from a patient treated with Natalizumab or directly Natalizumab reagent (Tysabri) was used as primary antibody in the immuno assay. Nucleus were stained with DAPI. Click here for file
