# Supplementary figures and images for: An immunoassay that distinguishes real neuromyelitis optica signals from a labeling detected in patients receiving natalizumab
Source: BMC Neurol. 2014 Jul 1;14:139. doi: 10.1186/1471-2377-14-139 (PMC4096525; doi:10.1186/1471-2377-14-139)

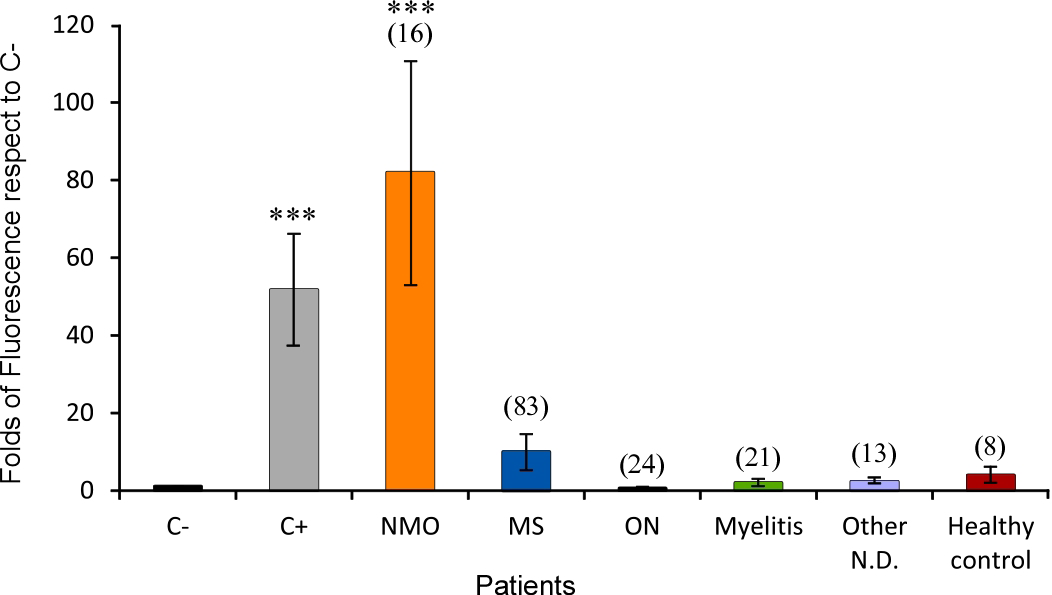

Supplement: Additional file 1: Figure S1 — Quantitative analysis from immunofluorescence assay. Serum from patients were used as primary antibody in an immunofluorescence assay. Quantification of fluorescence resulted from the densitometry analysis of fluorescence level using the NIH ImageJ software taking into account the fluorescent area. C+, corresponds to a positive control serum in which NMO-IgG were previously confirmed, and C-, corresponds with a negative control serum in which the Ab was absent. Quantification of fluorescence signal from serums of the six different groups was averaged together. Significant differences (*** p ≤ 0.001) respect to C- serum are indicated. Error bars are ± s.e.m (n=5). NMO: neuromyelitis; MS: multiple sclerosis; ON: optic neuritis; ND: neurological disorders. [file 1471-2377-14-139-S1.tiff]

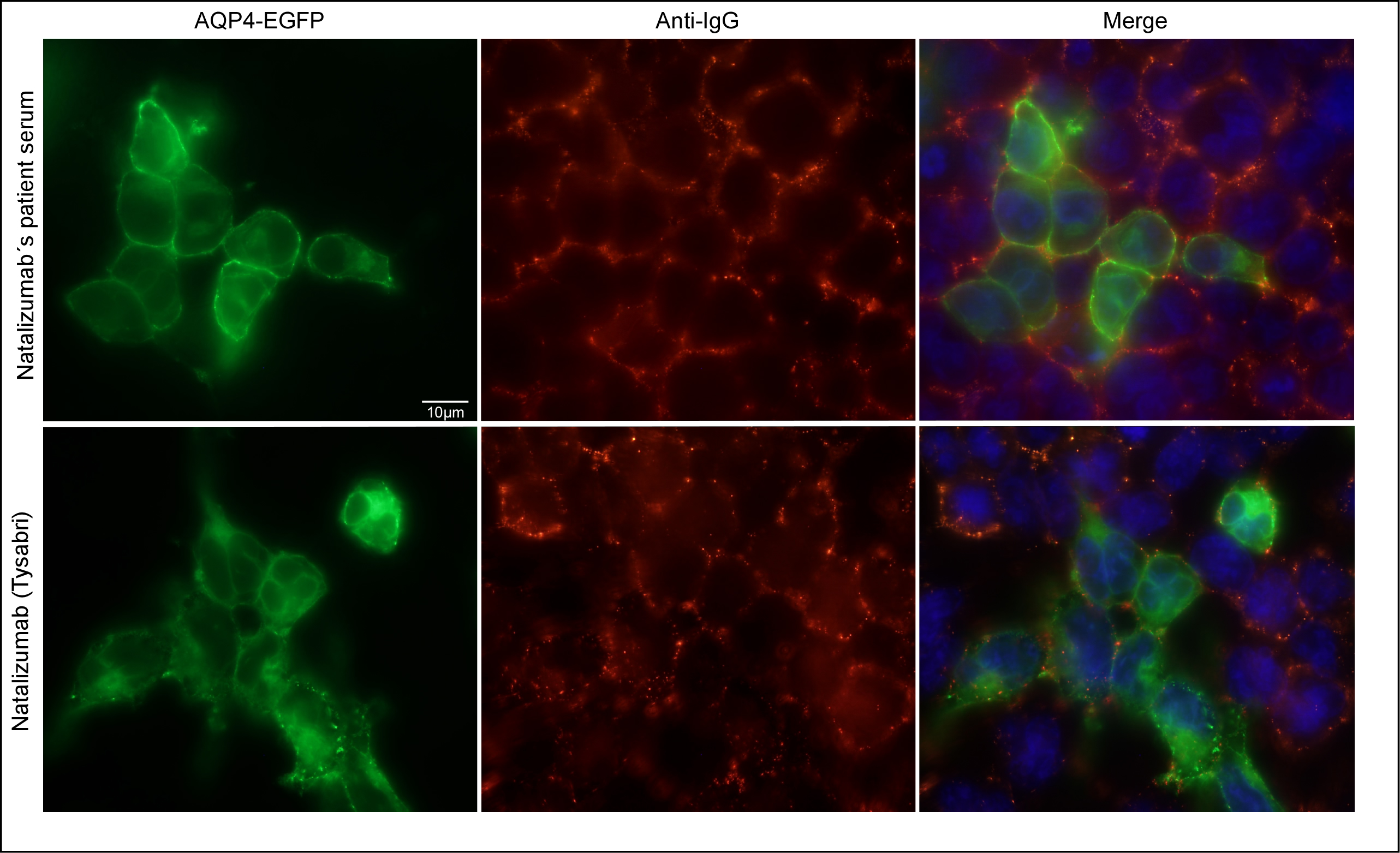

Supplement: Additional file 2: Figure S2 — Immunofluorescence assay using serum from natalizumab treated patient vs natalizumab reagent. Green fluorescent labeling was only detected in cells expressing EGFP-AQP4. However similar red fluorescence labeling was observed over all cells (expressing or not AQP4) when serum from a patient treated with Natalizumab or directly Natalizumab reagent (Tysabri) was used as primary antibody in the immuno assay. Nucleus were stained with DAPI. [file 1471-2377-14-139-S2.tiff]
